# Insights of COVID-19 pandemic impact on anesthetic management for patients undergoing cancer surgery in the National Cancer Institute, Egypt

**DOI:** 10.1186/s42077-020-00110-w

**Published:** 2020-11-11

**Authors:** Walaa Y. Elsabeeny, Omnia Y. Abd El Dayem, Ahmed Rabea, Rania S. M. Ibrahim, Heba G. M. Mahmoud, Eman Kamal, Randa A. Osman, Ayman Ghoneim

**Affiliations:** 1 Department of Anesthesia and Pain management, National Cancer Institute, Cairo University, Kasr Al Eini Street, Fom El Khalig, Cairo, 11796 Egypt; 2 Department of Clinical Pathology, Faculty of Medicine, Cairo University, 1 Alsaraya Street, Almanyal, Cairo, Egypt; 3 Department of Medical Oncology, National Cancer Institute, Cairo University, Kasr Al Eini Street, Fom El Khalig, Cairo, Egypt; 4 Department of Diagnostic and Intervention Radiology (Pediatric Unit), Faculty of Medicine, Cairo University, 1 Alsaraya Street, Almanyal, Cairo, Egypt; 5 Department of Surgical Oncology, National Cancer Institute, Cairo University, Kasr Al Eini Street, Fom El Khalig, Cairo, Egypt; 6 Department of Pulmonology, Faculty of Medicine, Cairo University, 1 Alsaraya Street, Almanyal, Cairo, Egypt; 7 Department of Clinical Pathology, National Cancer Institute, Cairo University, Kasr Al Eini Street, Fom El Khalig, Cairo, Egypt

## Abstract

**Abstract:**

New corona virus disease COVID-19 is a pandemic outbreak viral infection that is highly contagious. The disease can affect any age groups. Majority of patients show mild or no symptoms. Immunocompromised patients and patients with co-morbidities are more vulnerable to have more aggressive affection with higher rate of complications. Thus, cancer patients carry a higher risk of infection. Diseased patient can transmit infection throughout the disease course starting from the incubation period to clinical recovery. All healthcare workers contacting COVID-19-positive patients are at great risk of infection, especially the anesthesiologists who can be exposed to high viral load during airway manipulation. In the National Cancer Institute of Egypt, we apply a protocol to prioritize cases where elective cancer surgeries that would not affect patient prognosis and outcome are postponed during the early phase and peak of the pandemic till reaching a plateau. However, emergency and urgent surgeries that can compromise cancer patient’s life and prognosis take place after the proper assessment of the patient’s condition.

**Aim:**

This review aims to spot the management of cancer patients undergoing surgery during the COVID-19 pandemic in the National Cancer Institute, Egypt.

## Background

The emergence and spread of the new coronavirus disease 2019 (COVID-19) is considered a community crisis that threatens the world nowadays (Singhal 2020). The World Health Organization (WHO) has recently stated COVID-19 as a pandemic outbreak (https://www.who.int). All age groups are susceptible to infection by COVID-19, but a high rate of complications usually occurs with immunocompromised patients and older patients with co-morbidities (Singhal 2020; Chen et al. 2020a, 2020b). Cancer patients are considered immunocompromised, either due to their underlying malignancy or due to receiving chemotherapeutic agents as well as radiotherapy; thus, they carry a higher risk of infection that can reach two folds that in the normal healthy population (Al-Shamsi et al. 2020).

The disease may present by variable clinical manifestations like fever, cough, shortness of breath, sore throat, rhinorrhea, headache, chest pain, diarrhea, nausea, and vomiting (Chen et al. 2020a, 2020b). The virus shows human to human transmission (Chan et al. 2020) mainly through droplet infection either by inhalation of aerosol droplets or touching a surface contaminated by the virus and retouching the mouth, nose, or eyes (Singhal 2020). The main diagnostic laboratory tool for COVID-19 is reverse transcription-polymerase chain reaction (RT-PCR) with a high sensitivity which varies according to the specimen type (Lippi et al. 2020; Zhao et al. 2020). Laboratory data usually displays lymphopenia; prolonged prothrombin time (PT), with the elevation of alanine transferase (ALT); D-dimer; lactate dehydrogenase (LDH); and C-reactive protein (CRP) (Wang et al. 2020a, 2020b, 2020c). Chest computed tomography (CT) plays an important role in confirming the diagnosis in addition to monitoring of disease course (Xie 2020). In this review, a comprehensive understanding of the diagnostic imaging, key imaging findings, atypical features, and evolution of chest imaging findings associated with disease progression or clinical improvement is made for effective cancer patient management especially those undergoing surgical intervention.

Diseased patients can be asymptomatic (He et al. 2020) at the time of surgery which necessitates dealing with each patient as a potential COVID19 case (He et al. 2020). Health care workers are at risk of infection (Lai et al. 2020). Manipulating the airway of a COVID-19 patient carries a high risk of infection to the anesthesiologist, due to the viral shedding in aerosol respiratory secretions during intubation.

In areas that have been considered endemic, elective cancer surgeries that can be delayed without threatening the patient’s life or prognosis should be postponed (Liang et al. 2020). However, this can be applicable during the peak of the disease till reaching a plateau “which is a state of a relative stable level of infection rates.” If the plateau is extended until finding a definitive treatment or a vaccine, the scope of surgeries can be widened to be included. In Egypt, the earliest disease predicted peak timing estimated by researchers is by mid of May 2020 and the latest could be by mid of July 2020 (El Desouky 2020).

## COVID-19 epidemiology

In December 2019, a highly contagious rapidly spreading, severe acute respiratory syndrome coronavirus 2 (SARS-CoV-2) was first discovered in Wuhan city, China (Wang et al. 2020a, 2020b, 2020c). Initial cases were reported following the exposure to a live animal trading market with theories suggesting that the disease was initially transmitted from bats to humans (Singhal 2020; Benvenuto et al. 2020). Thereafter, cases not related to live animal markets started to appear denoting the capability of the virus to be transmitted from human to human (Huang et al. 2020a, 2020b). All age groups are susceptible to infection. Children and most of the healthy adult patients present with flu-like symptoms (Benvenuto et al. 2020). Infection can be transmitted during the incubation period (which can extend from 2 to 14 days) and from symptomatic, asymptomatic patients as well as patients on clinical recovery (Singhal 2020; Chan et al. 2020; He et al. 2020; C. Huang et al. 2020a, 2020b; Ye et al. 2020). Disease transmission commonly occurs through nasal and oral droplet infection, with fecal-oral transmission as a less common possible route. Another opinion suggests its capability of transmission through aerosols during prolonged close contact (Singhal 2020; Rothe et al. 2020; Ye et al. 2020).

## Cancer patients and COVID-19

Cancer patients are more prone to be infected by COVID-19 with unfavorable prognosis (Al-Shamsi et al. 2020). They are considered immunocompromised either due to their underlying malignancy or due to receiving chemotherapeutic agents as well as radiotherapy. Extrapolating the data from a retrospective study in 2009 regarding Influenza A (H1N1), cancer patients had a higher risk for pneumonia (66%) and 30-day mortality of 18.5% (Dignani et al. 2014). Therefore, the special attention should be given to these populations. There are some special recommendations for stable cancer cases in endemic areas, elective cancer surgeries and adjuvant chemotherapy should be postponed, and cancer patients and cancer survivors should be well educated about proper personal protection.

The diagnosis and the proper intervention for new cases should not be compromised during the pandemic so as not to delay the treatment nor compromise the early stage of the disease; a study published by an American group showed that delaying the treatment or postponing it has increased the mortality of cancer patients (Mehta et al. 2020) and it will definitely affect the survival of cancer patients. Proper screening methods should be strictly applied to newly diagnosed patients in the outpatient clinics, and the medical team should be properly aware and strictly adherent to the proper protection through handwashing, personal protective equipment (PPE), and changing the medical gloves between each patient.

For patients who are on treatment, proper spacing between the chemotherapy sessions should be applied and if the protocol allows, extend the duration between cycles. Medications that could be dispensed remotely should be encouraged after contacting the patient over the phone and assuring his/her safety and adherence to his/her medication. This will also add an element of psychological support and security that the medical team is supporting him/her. If the patient must come to the outpatient clinics, then the proper protection for the patient must be applied through strict handwashing, facial mask protection, and social distancing of at least 1.5 m.

Patients who finished their treatment and are under follow-up could be assessed over the phone through a questionnaire and not to be seen in the outpatient clinics, to avoid their exposure to suspected cases, thus protecting them and clearing the hospitals to deal with the more urgent cases during this pandemic. In addition, a closer observation and aggressive management should take place particularly in elder cancer patients with comorbidities (Liang et al. 2020). Also, we have increased the follow-up time interval to allow less hospital visits and minimize the workload on the medical team to allow proper handling of the suspected COVID-19 cases.

## Diagnosing cancer patient with COVID-19

### Clinical manifestations

Clinical presentations range from asymptomatic or mild symptoms to severe disease-causing death (https://www.cdc.gov/) (Hui et al. 2020). In an initial report of 41 patients infected in Wuhan, China, Huang et al. reported that the most common clinical findings were fever (98%), followed by cough (76%) and myalgia/fatigue (44%). Headache, sputum production, and diarrhea were less common. The clinical course was characterized by the development of dyspnea in 55% of patients and lymphopenia in 66% (Huang et al. 2020a, 2020b). Symptoms may develop from 2 days up to 2 weeks following exposure to the virus. The mean incubation period was 5.1 days and 97.5% of individuals who developed symptoms did so within 11.5 days of infection (Lauer et al. 2020) ( https://www.cdc.gov/).

### Laboratory features

The current gold standard for the diagnosis of SARS-CoV-2 infection is (real-time) reverse transcription-polymerase chain reaction (RT-PCR) on respiratory tract specimens (Lippi et al. 2020). Unfortunately, the quality of RT-PCR testing could be jeopardized by many factors. Some of these are common to other diagnostic areas (e.g., identification errors, collection, handling, and storage of the specimen, sample quality, and performance of the assay or of the equipment), whilst others are very specific (e.g., virus-specific diagnostic window, sample contamination, incorrect nucleotide incorporation, non-specific PCR annealing, sample type) (Lippi et al. 2020). These problems might significantly lower the sensitivity of the detection and lead to a noteworthy delay of early diagnosis and management of patients (Zhao et al. 2020). However, RT-PCR remains the most useful laboratory diagnostic test for COVID-19 worldwide.

Another most widely used diagnostic method is a serological test for the presence of antibodies against viral proteins. Immunoglobulin M (IgM) is usually the first responding antibody eliminating pathogens before sufficient immunoglobulin G (IgG) is produced, while IgG serves as the most robust antibody-based immunity (Zhang et al. 2020a, 2020b). It was shown that in the early phase of illness, within 7 days of onset, the ribonucleic acid (RNA) test has the highest sensitivity that differs according to sample type as described by Wang et al.; bronchoalveolar lavage fluid specimens showed the highest positive rates (93%), followed by sputum (72%), nasal swabs (63%), fibro-bronchoscope brush biopsy (46%), pharyngeal swabs (32%), feces (29%), and blood (1%). None of the urine specimens tested positive (Wang et al. 2020a, 2020b, 2020c).

Despite the fact that the antibody assays in the early illness phase presented a positive rate of 38.3%, antibody sensitivity overtook that of RNA test since day 8 after onset and reached over 90% across day 12. Thus, the combined use of RNA and antibody testing improved markedly the sensitivities of pathogenic diagnosis for COVID-19 patients in different phases (Zhao et al. 2020).

The most common abnormal laboratory changes noticed in the affected patients were lymphopenia with depletion of CD4 and CD8 lymphocytes, prolonged prothrombin time, elevated lactate dehydrogenase, elevated D-Dimer, elevated alanine transaminase, C-reactive protein, and creatinine kinase (Wang et al. 2020a, 2020b, 2020c). Laboratory abnormalities may indicate the severity of disease and developing complications. Most patients with secondary infection had a procalcitonin level greater than 0.5 ng/ml, and intensive care unit (ICU) patients had higher levels of prothrombin time and D-dimer (Huang et al. 2020a, 2020b). Also, hypoalbuminemia, lymphopenia, high concentrations of CRP, and elevated LDH predict the severity of acute lung injury. Higher levels of angiotensin II are also proposed to be related to acute lung injury (Liu et al. 2020a, 2020b). Meanwhile, non-survivors are suggested to be those who had higher levels of D-dimer and FDP, longer PT and a PTT, and lower fibrinogen and antithrombin levels (Tang et al. 2020).

In general, poor prognostic indicators for infected patients include neutrophil to lymphocyte ratio more than 3.13, absolute lymphocyte count less than 0.8, LDH more than 245 U/L, ferritin more than 300ug/L, CRP more than 100 mg/L, and D-dimer more than 1000 ng/ml (https://journals.lww.com/em-news/blog/breakingnews/pages/post.aspx?PostID=508) (Liu et al. 2020a, 2020b).

Concerning cancer patients, who are regarded as a highly vulnerable group in the current (COVID-19) pandemic, it was reported that they present with similar features to the general population, except for anemia and hypoproteinemia, which are more frequently found in this cohort. Both could be considered a major consequence of nutritional deterioration in cancer patients, which may adversely affect immunocompetence and increase the susceptibility to respiratory pathogens (Zhang et al. 2020a, 2020b).

### Radiological features

Because of the primary involvement of the respiratory system in COVID-19 patients, recent studies addressed the diagnostic value of chest computed tomography (CT) examination especially with an initial false-negative reverse transcription-polymerase chain reaction (RT-PCR) results (Xie 2020; Huang et al. 2020a, 2020b). CT examination is of great significance not only in diagnosing COVID-19 but also in monitoring disease progression and evaluating therapeutic efficacy (Xie 2020).

Chest radiographs are of little diagnostic value in early stages, as it has a high specificity of up to 90% but a low sensitivity of about 25% for detection of COVID-19-related lung opacities, whereas CT findings may be present even before symptom onset (Huang et al. 2020a, 2020b). However, in the first 4 days of infection with the SARS-cov-2 virus, the CT is not very sensitive, after that the CT has a high sensitivity of up to 98% but moderate to low specificity between 25 and 56% (Fang et al. 2020).

A wide variety of CT findings in COVID-19 was reported in several studies. After combining the available data in the previous literatures, it was found that the characteristic patterns and distribution of initial CT manifestations as ground-glass opacification (GGO) (88%), bilateral involvement (88%), posterior distribution (80%), multi-lobar involvement (79%), and peripheral distribution (76%); however, consolidative opacities (32%) mainly present in the elderly population (Xie 2020). Other CT findings include interlobular septal thickening, bronchiectasis, pleural thickening, and subpleural involvement, with various rates across the studies, they present mainly in the later stages of the disease (Kay and Abbara 2020; Shi et al. 2020). Pleural effusion, pericardial effusion, lymphadenopathy, cavitation, CT halo sign, pneumothorax, multiple minute pulmonary nodules, and tree in bud appearance are uncommon or even rare findings. Therefore, the presence of these findings should raise the possibility of other diagnoses rather than COVID-19 disease or may be seen with disease progression (Song et al. 2020; Ai et al. 2020). Hence, CT can play a role in the triage of cancer patients undergoing surgical intervention, it can exclude or diagnose the possibility of COVID-19 infection, it determines the severity of the disease, and it plays a role in predicting the worsening or improvement of the disease.

### Prioritization of cancer surgeries during COVID-19 pandemic

Elective cancer surgeries not affecting patient survival or disease prognosis are postponed during the peak and allowed during the plateau for proven COVID-19-free patients. Surgical treatment of cancer patients should be time sensitive with prioritization of emergency cases during the pandemic peak when all the health care workers and resources are consumed in the management of COVID-19-positive patients (Schrag et al. 2020). We follow special guidelines for different subspecialties in surgical oncology. Avoiding major resections and complex anastomoses should be the rule. Surgeons should manage patients with the least surgical procedures to avoid a long postoperative hospital stay and to minimize complications. Precautions to protect health care workers should be taken including wearing all available PPE (personal protective equipment).

### Elective cases

Patients undergoing elective surgery should be screened for COVID-19, and surgery should be deferred in COVID-19-positive patients (Aminian et al. 2020).

### Emergency cases

Admission of the emergency cases for surgery should follow the general diagnostic rules outlined in this article to diagnose COVID-19. During the pandemic crisis, any patient admitted to the cancer center should be considered a COVID-19-positive patient until proven otherwise. These cases may include:

- *Airway obstruction*: Airway compromise needing tracheostomy like laryngeal cancers, post-cricoid carcinoma, and post thyroid surgery vocal cord paralysis or hematoma.
- *Bowel obstruction*: Emergent cancer cases presenting with intestinal obstruction from colon cancer or any other cause that cannot be managed except by surgery is a priority (Gallo et al. 2020).
- *Bleeding*: Bleeding from the gastrointestinal tract requiring emergency intervention and postoperative bleeding (Gallo et al. 2020).
- *Postoperative complications*: Burst abdomen following abdominal procedures. Any postoperative complication that needs surgical interventions like high output fistulae and major leaks from GI anastomoses.

### Urgent cases

The most urgent cases are those patients who cannot be given chemo or radiotherapy (tumor not sensitive or will result in major complications), and surgery is their primary mode of treatment and postponing their surgeries would likely affect their survival or results in the advancement of their cancer stage or significantly affect their outcome.

According to tumor site:

- *Breast cancer*: Surgery is considered urgent for cases of malignant phyllodes, patients with progressive disease on chemotherapy, and angiosarcoma (Bartlett et al. 2020).
- *Hepatobiliary and pancreatic tumors*: Surgery should be considered for patients with aggressive hepatobiliary and pancreatic tumors including colorectal cancer metastatic to the liver, cholangiocarcinoma, gastric, duodenal, and ampullary cancer (Bartlett et al. 2020).
- Colorectal cancer: Early stage non-metastatic colon cancer should be operated upon as well as early stage rectal cancer. Transfusion-dependent colorectal cancers should undergo surgery urgently (Gallo et al. 2020).
- Gastroesophageal cancers: Gastrointestinal stromal tumor (GIST) should undergo urgent surgery if the patient is symptomatic or has bleeding. Very early stages of gastric cancer that can undergo endoscopic resection are better to be managed by endoscopy if available. Early gastric cancers should be managed by surgery (Bartlett et al. 2020).

Endocrine tumors:

- *Thyroid gland*: Rapidly progressive tumor invading the airway or vocal cords (Bartlett et al. 2020).
- *Para thyroid gland*: Life-threatening hypercalcemia secondary to hyperparathyroidism not controlled through medical treatment (Bartlett et al. 2020).
- Adrenal gland: Functional adrenal tumors not responding to medical treatment, e.g., pheochromocytoma (Bartlett et al. 2020).
- Soft tissue tumors: Non-metastatic soft tissue sarcoma under staging.

### Anesthetic management

Hospitalized patients are at a greater risk for nosocomial infection, thus should receive special attention (Lai et al. 2020). Unless proved negative (−ve) COVID-19 by laboratory and radiological findings, any patient present to surgery can be a diseased asymptomatic COVID-19 patient (He et al. 2020), thus necessitates dealing with such patient as a potentially infected one. Highly suspected cases to have COVID-19 with no time to do the test (due to urgency) should be treated as those confirmed cases with COVID-19 (Li et al. 2020). High-risk cases should be discussed with surgeons regarding the degree of urgency of operation, and the risk benefits if a delay is decided.

**Fig. 1:**
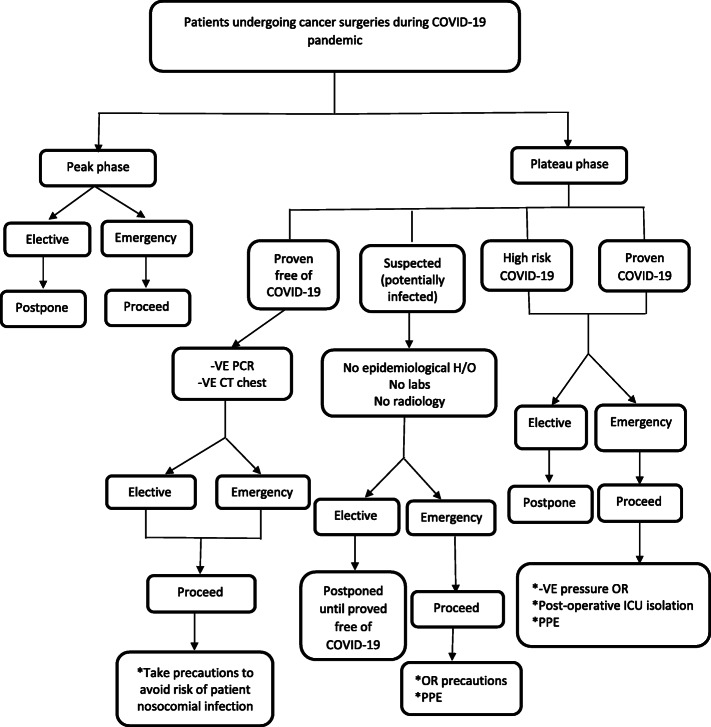
Patients undergoing cancer surgeries during COVID-19 pandemic

The patient is considered highly suspected COVID-19 if there is a history of traveling from high-risk areas, contact with cases of proved COVID-19 or contact with patients who have fever or respiratory symptoms within 14 days, and clustered cases onset, in addition to at least two clinical presentations of fever and/or respiratory symptoms, radiological features of pneumonia, a normal or decreased (white blood cell) WBC count, normal or decreased lymphocyte count in the early stage of onset (Jin et al. 2020), (http://www.satcm.gov.cn/), Fig. 1.

## Preoperative evaluation

The main focus in preoperative assessment is to identify high-risk patients and procedures, in order to optimize the patient’s respiratory condition as appropriate. The infection control team should be involved early in suspected cases considering a prompt request for a rapid test to confirm the diagnosis to guide the managing team.

### Elective cases

Health care workers are at risk of infection (Lai et al. 2020). Strict precautions should be followed throughout patient assessment in the preoperative assessment clinic. The responsible anesthetist and healthcare workers should wear gowns, disposable gloves, surgical or fit N95 masks, and eye goggles or face shield. Careful handwashing between patients should be adopted. Any patient suspected of COVID-19-positive should be reported to the infection control team (Chen et al. 2020a, 2020b).

### Emergency cases

The responsible anesthetist in full personal protective equipment (PPE) will assess the patient as follows: assessment and evaluation of respiratory status through checking arterial blood gasses, X-ray, and/or CT chest radiography; full assessment of airway; and assessment for signs of shock or organ failure. Patients can present at the time of assessment with fever, chest crepitations, wheezing, and desaturation. Complete blood count, CT chest, liver function, and renal function tests should be requested.

### Choice of anesthesia

General or regional anesthesia can be performed according to the site of surgery and patient’s condition, provided normal coagulation profile, platelet count, and function.

Performing regional anesthesia is better adopted whenever possible, thus avoiding airway manipulation and consequently reducing exposure to aerosols during coughing (Lie et al. 2020).

### Intraoperative management

Using negative pressure operating theater is preferable to positive pressure one in COVID-19 highly suspected or confirmed cases to minimize the risk of viral spread (Wax and Christian 2020).

The main concern is to minimize as possible the number of health care providers exposed to the infected patient, and protect all of them as well as the patient through lowering the number of personnel in the operating room during the induction of anesthesia and throughout all the time the patient is in the theater, applying a bacterial viral filter to the expiratory limb of the breathing circuit, and applying disposable covers to surfaces to minimize contaminations (Wax and Christian 2020). All health care providers in the theatre should wear personal protective equipment (PPE). They should first have appropriate hand hygiene and then wear a fit N95 respirator, face protector shield, gown, and gloves (Velly et al. 2020).

### Induction of anesthesia

Standard monitoring and pre-check intravenous access, instruments, drugs, ventilator, and suction should be applied (Orser 2020). Infection control measures should be strictly followed when intubating a COVID-19 patient, in order to avoid exposure to high viral load (Wang et al. 2020a, 2020b, 2020c) and preoxygenation with 100% oxygen at the minimal possible gas flow to ensure a good seal with a face mask. Intubation is better to be done by video laryngoscopy (Wax and Christian 2020). The use of video laryngoscopes aids to perform the first attempt easy, to keep a distance away from the patient’s airway, and to ensure complete muscle paralysis before endotracheal intubation to avoid cough (Lingappan et al. 2018).

Intubation should be done by an expert anesthesiologist in order to minimize the time of intubation and number of attempts (Wax and Christian 2020). Unless indicated, awake fiberoptic intubation should be avoided as atomized local anesthetic use can cause viral aerosolization and spread (Orser 2020).

Rapid sequence induction after a full dose of muscle relaxant is required to reduce the need for bag-mask manual ventilation of the lungs with the potential aerosolization of the virus from the patient’s airways (Li et al. 2020; Wax and Christian 2020; Caputo et al. 2006). If manual ventilation is anticipated, small tidal volumes should be applied (Kamming et al. 2003) with the use of a two-hand grip technique to ensure a good seal with a face mask. During induction of anesthesia if fentanyl is used, it should be administered slowly and preceded by 0.5 mg/kg lidocaine to suppress cough (Tan et al. 2018).

Apply positive pressure ventilation after inflation of the endotracheal tube cuff and confirm the endotracheal tube in position by observing bilateral chest wall movement and by CO_2_ curve on capnography, as auscultation may be difficult due to the PPE (Wax and Christian 2020).

### Maintenance of anesthesia

Apply lung-protective mechanical ventilation strategy through setting the low tidal volume at 6 ml/kg and adjusting the respiratory rate to maintain minute ventilation and normo-capnea. Peak airway pressure should be noticed to be below 30 mmHg (Fan et al. 2018). During the mechanical ventilation, minimize the circuit disconnection to avoid aerosol dissemination in the environment and try to use a closed suctioning system when necessary.

### Emergence from anesthesia

Smooth emergence should be the plan to avoid coughing and using anti-emetics to avoid vomiting. The patient should be kept after emergence in an isolation operating theater and arrange for case handover with the receiving nurse in the operating theater (Li et al. 2020). Administering high flow oxygen, nebulized medications, or noninvasive ventilation should be avoided (Tan 2004).

After the end of surgery, medical health providers should follow the instructions for taking off their PPE with proper hand hygiene to avoid self-contamination (Li et al. 2020; Peng et al. 2020). Disposable equipment should be discarded, and anesthesia machines and monitors should be properly disinfected under the supervision of a member of the infection control team.

## Conclusion

The new coronavirus disease 2019 (COVID-19) emerged as a crisis that was stated to be a pandemic by WHO. Cancer patients are more vulnerable to get infected with more morbidity and mortality. Judgmental evaluation and management of each cancer patient should be considered during the pandemic especially during the peak of the disease. Elective cancer surgeries that can be delayed without threatening the patient’s life or prognosis should be postponed till the plateau is reached; however, emergency and urgent surgeries that can affect the patient’s life or prognosis should take place. All healthcare workers especially the anesthetists are at great risk for infection. Full precautions and strict adhesion to infection control measures must be adopted.

## Data Availability

Not applicable

## Abbreviations

COVID-19: Coronavirus disease 2019
WHO: World Health Organization
RT- PCR: Reverse transcription-polymerase chain reaction
PT: Prothrombin time
ALT: Alanine transferase
LDH: Lactate dehydrogenase
CRP: C-reactive protein
CT: Computed tomography
SARS-COV2: Severe acute respiratory syndrome coronavirus 2
H1N1: Influenza A
PPE: Personal protective equipment
PCR: Polymerase chain reaction
IgM: Immunoglobulin M
IgG: Immunoglobulin G
RNA: Ribonucleic acid
CD 4: Cluster of differentiation 4
CD 8: Cluster of differentiation 8
ICU: Intensive care unit
FDP: Fibrin degradation product
aPTT: Activated partial thromboplastin time
WBC: White blood cell count
SPO_2_: Oxygen saturation
CO_2_: Carbon dioxide
